# The role of sarcomere length non-uniformities in residual force enhancement of skeletal muscle myofibrils

**DOI:** 10.1098/rsos.150657

**Published:** 2016-03-30

**Authors:** Kaleena Johnston, Azim Jinha, Walter Herzog

**Affiliations:** Human Performance Laboratory, Faculty of Kinesiology, University of Calgary, Alberta, Canada T2N 1N4

**Keywords:** residual force enhancement, sarcomere length non-uniformity, myofibril, history dependence, descending limb of the force–length relationship, sarcomere popping

## Abstract

The sarcomere length non-uniformity theory (SLNT) is a widely accepted explanation for residual force enhancement (RFE). RFE is the increase in steady-state isometric force following active muscle stretching. The SLNT predicts that active stretching of a muscle causes sarcomere lengths (SL) to become non-uniform, with some sarcomeres stretched beyond actin–myosin filament overlap (popping), causing RFE. Despite being widely known, this theory has never been directly tested. We performed experiments on isolated rabbit muscle myofibrils (*n* = 12) comparing SL non-uniformities for purely isometric reference contractions (I-state) and contractions following active stretch producing RFE (FE-state). Myofibrils were activated isometrically along the descending limb of the force–length relationship (mean ± 1 standard deviation (SD) = 2.8 ± 0.3 µm sarcomere^−1^). Once the I-state was reached, myofibrils were shortened to an SL on the plateau of the force–length relationship (2.4 µm sarcomere^−1^), and then were actively stretched to the reference length (2.9 ± 0.3 µm sarcomere^−1^). We observed RFE in all myofibrils (39 ± 15%), and saw varying amounts of non-uniformity (1 SD = 0.9 ± 0.5 µm) that was not significantly correlated with the amount of RFE, but through pairwise comparisons was found to be significantly greater than the non-uniformity measured for the I-state (0.7 ± 0.4 µm). Three myofibrils exhibited no increase in non-uniformity. Active stretching was accompanied by sarcomere popping in four myofibrils, and seven had popped sarcomeres in the I-state. These results suggest that, while non-uniformities are present with RFE, they are also present in the I-state. Furthermore, non-uniformity is not associated with the magnitude of RFE, and myofibrils that had no increase in non-uniformity with stretch still showed normal RFE. Therefore, it appears that SL non-uniformity is a normal associate of muscle contraction, but does not contribute to RFE following active stretching of isolated skeletal muscle myofibrils.

## 1. Introduction

Residual force enhancement (RFE) is a property of skeletal muscle that is defined as the excess amount of steady-state force exhibited during an isometric contraction following an active stretch, compared with the force observed during a purely isometric contraction at the same length, for a given level of activation [1,2]. RFE introduces a conundrum into the current theory of skeletal muscle contraction, the cross-bridge theory [3–6], because it distinctly violates the force–length relationship [4,7–9], because muscles contracting at the same length produce vastly different isometric steady-state forces, depending on their contractile history. Due to the inability to account for RFE with the cross-bridge theory [10], extensive research has been conducted at all structural levels of muscle with the aim of elucidating the mechanisms responsible for RFE [1,2,11–20]. Combined, the observations from these studies suggest that RFE is a property that originates in the basic contractile unit of skeletal muscle, the sarcomere [16]. For this reason, isolated myofibrils, in which all sarcomeres are arranged strictly in series and their instantaneous forces and variable lengths can be measured continuously, make the perfect preparation for studying the characteristics of RFE [14,17,21–26].

A popular mechanism thought to be responsible for RFE is the sarcomere length non-uniformity theory (SLNT) [24,25]. Proponents of the SLNT suggest that upon active stretching along the descending limb of the force–length relationship, muscles develop vast sarcomere length non-uniformities that are not present for purely isometric contractions. These non-uniformities are thought to occur because of instability of sarcomeres on the descending limb of the force–length relationship [7], which causes most of the stretch to be taken up by initially slightly longer, and therefore weaker, sarcomeres, while the initially short, and thus strong, sarcomeres remain at about a constant length [24,25]. This causes the long/weak sarcomeres to be stretched to lengths beyond actin–myosin filament overlap (they are said to ‘pop’), and their force originates from passive structural elements of muscle exclusively [24,25]. Once the final steady-state force following active stretching is reached, the passive forces of the ‘popped’ sarcomeres match the active forces of the sarcomeres that remain approximately at the initial pre-stretch length [24,25]. The SLNT suggests that this process occurs with sequentially weaker sarcomeres popping until the end of the stretch, at which point the sarcomere length (SL) distribution along the myofibril is non-uniform. The non-uniformity in a myofibril (serially arranged sarcomeres) is characterized by SLs in two distinct groups: one set of sarcomeres at a specific point on the descending limb relying primarily on active force, and the other set at a specific length beyond actin–myosin filament overlap relying exclusively on passive force. These non-uniformities cause the myofibril to produce force proportional to the filament overlap for the shorter sarcomeres, which is greater than what is predicted from an isometric contraction with (assumed) uniform SL, thus producing the experimentally observed RFE.

The SLNT has been expressed as a mathematical model, which incorporates predictions regarding RFE following active stretch of muscle [24,25]. The first prediction of the SLNT is that shorter/stronger sarcomeres in a purely isometric contraction (I-state) will lengthen less than longer/weaker sarcomeres during active stretch. From this, longer SLs in the I-state should be associated with a greater length change during active stretch. This leads to the second prediction of the SLNT which is that the SL distribution is highly non-uniform in an isometric contraction following active stretch (FE-state) while it is essentially uniform in the I-state [24,25]. The third prediction of the SLNT is that active stretching results in sarcomeres being pulled to lengths beyond actin–myosin filament overlap. Finally, the forth prediction is that due to the sequential nature of the popping of individual sarcomeres, as described by the SLNT, an increase in stretch magnitude should result in a greater number of popped sarcomeres, causing the SL distribution to become more non-uniform with increasing stretch magnitude. Because RFE is known to increase with the magnitude of active stretching [1,2,12,20], an increase in RFE should be associated with an increase in SL non-uniformity.

Despite extensive research regarding RFE in skeletal muscle, the detailed and specific assumptions and predictions of the SLNT have not been tested directly. Specifically, SL changes (prediction 1) and distributions (prediction 2) have not been quantified and directly compared for the I- and FE-states in the same myofibril. In addition, the idea that sarcomeres pop during active stretching (prediction 3), and not during isometric contractions, even if the isometric contractions occur on the descending limb of the force–length relationship, has not been tested systematically [27–29]. Furthermore, changes in SL non-uniformity have not been correlated to the amount of RFE (prediction 4). Therefore, the purpose of this study was to experimentally test the basic assumptions and predictions of the SLNT by comparing SL distributions obtained in isolated myofibrils during isometric contractions pre- (I-state) and post- (FE-state) active stretch at the same length and activation. In accordance with the SLNT, we hypothesized that (i) longer individual SL in the I-state correlates positively with individual SL change between the I- and FE-states; (ii) that myofibrils in the FE-state have greater SL non-uniformity than the I-state; (iii) that active stretching is always accompanied by the popping of some sarcomeres; and (iv) that an increase in SL non-uniformity correlates positively with RFE.

## 2. Methods

### 2.1. Experimental protocol

Eight six-month-old female New Zealand White rabbits were euthanized by an intravenous injection of sodium pentobarbital according to a protocol approved by the Life and Environmental Sciences Animal Care Committee of the University of Calgary. Isolation of single myofibrils was accomplished as previously published [14,18,21,26,30,31]. Experiments were conducted using a 100× oil immersion objective in phase-contrast illumination (numerical aperture 1.3). A Rolera Bolt^®^ camera (Quantitative Imaging Corp., Surrey, Canada) attached to the microscope was used to record all of the experiments on StreamPix 5 video imaging software (NorPix Inc., Montreal, Canada) at 30 Hz (optical resolution: 87 nm pixel^−1^, functional resolution: 0.1 µm pixel^−1^). Myofibril length changes were delivered through a custom-written software program (LabVIEW^®^, National Instruments Corp., Austin, TX) that controlled radial piezo-tubes (part no. PZT-5H with 90° quadrants, Boston Piezo-Optics Inc., Bellingham, MA) that held the needle, which was attached to the myofibril at one end. The myofibrils were attached to one of a pair of cantilevers for force measurement at their opposite end.

**Figure 1.**
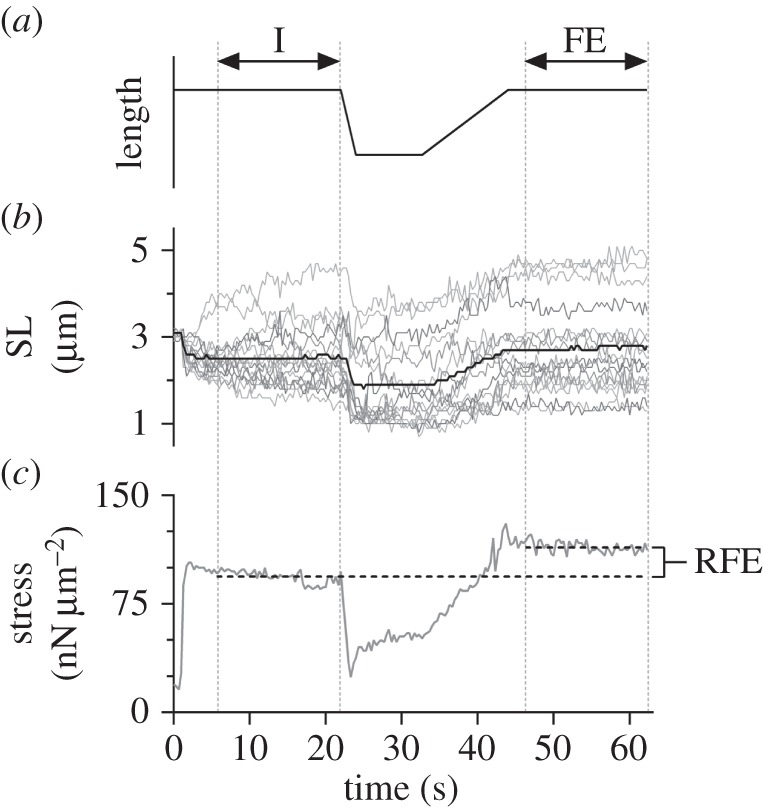
(*a*) Motor (needle) movement as a function of time for the experimental protocol. Note that the stretch and shortening times varied based on the number of sarcomeres along each myofibril, but the overall pattern of movement was identical for all 12 myofibrils. (*b,c*) SL and stress as a function of time for myofibril 5. (*b*) The thick black line represents the average sarcomere length throughout the experiment. The thin grey lines represent the individual sarcomeres (*n* = 16). (*c*) Horizontal dashed black lines depict the average stress in the I- (bottom) and FE- (top) states, with the corresponding amount of RFE (22%) indicated. Vertical grey dotted lines through (*a–c*) identify the I- and FE- steady states.

Myofibrils (*n* = 12) with a good striation pattern (defined as visible I-bands, A-bands and Z-lines) and 5–23 sarcomeres in length, were activated via a specialized jetted fluid (Jacuzzi) technique at a mean SL of 2.8 ± 0.3 µm. The Jacuzzi delivered the calcium-based activating solution (room temperature; see ‘Solutions’) directly to the myofibrils. The myofibrils reached the I-state contraction prior to being rapidly shortened (0.8 µm s^−1^ sarcomere^−1^) to an average SL of 2.4 µm, where they were held for 10 s. The myofibrils were then slowly stretched (0.1 µm s^−1^ sarcomere^−1^) back to a mean length of 2.9 ± 0.3 µm sarcomere^−1^ and allowed to reach the FE-state (figure 1*a*). The active shortening phase of the experimental protocol brings the sarcomeres to the initial length required prior to active stretch. By performing this shortening step quickly and following it by a 10 s pause, any history-dependent effects are gone, and so the protocol is equivalent to deactivating the myofibril, bringing it back to the initial length and then reactivating it [2,32], a protocol much more prone to errors than the one used.

### 2.2. Analysis

Individual SLs were defined as the distance between the centroids of adjacent A-bands, as identified using a custom-written MATLAB^®^ software program (The MathWorks Inc., Natick, MA) [14,18,19,26,30,33–36]. The software program was also used to track the displacement of the attached cantilever relative to the unattached, reference cantilever. Force was calculated by multiplying the displacement of the cantilever from its zero force reference position by its stiffness (132 nN µm^−1^). Forces were normalized to the cross-sectional area, calculated using the diameter of each myofibril at optimal length (2.4 µm sarcomere^−1^) and expressed in units of stress (nN µm^−2^) [14,16,22,26,30,31,33–40]. Analysis of every 10th video frame (i.e. a nominal frequency of 3 Hz) allowed for the generation of time history graphs for individual SLs and stress, which were used to identify I- and FE-states (figure 1*b*,*c*). The movement of the cantilever that allowed for the quantification of force resulted in small length changes in the myofibril upon activation; therefore, the I-state was defined as the time point following activation at which the mean SL and the myofibril stress were constant. Constant stress was determined visually from the force–time graph as the point when the total myofibril length and therefore, force reached a plateau following activation. The FE-state was defined similarly, following myofibril lengthening until the end of the protocol. Mean SL, standard deviation (SD) of the mean SL, and stress were determined for the I- and the FE-states for each myofibril. The SD of the mean SL was used to quantify the amount of SL non-uniformity, whereby an increase in SD was indicative of a more non-uniform distribution [34,36]. A Wilcoxon signed ranks non-parametric test was performed to compare stress in the I- and FE-states to determine if there was a significant increase in force. In order to test the first prediction of the SLNT, a Pearson *R* correlation was used to determine the relationship between individual SL in the I-state and the length change between the I- and FE-states. The second prediction of the SLNT was tested using a Wilcoxon signed ranks non-parametric test to compare SL non-uniformity (SD) in the I- and FE-states. The third prediction was analysed by determining individual SLs in each state to identify which sarcomeres had popped beyond myofilament overlap (SL ≥ 4.0 µm). A Fisher's exact test was then performed to determine if there was a difference in the number of popped sarcomeres between the I- and FE-states. The fourth prediction was analysed using a Spearman *R* correlation to identify the relationship between the increase in SD between the I- and FE-states and the amount of RFE. A significance level of 0.05 was used for all of the statistical tests.

### 2.3. Solutions

Rigor solution: Tris (50 mM), sodium chloride (100 mM), potassium chloride (2 mM), magnesium chloride (2 mM) and ethylene glycol bis(2-aminoethyl ether)-*N,N,N*′*N*′-tetraacetic acid (EGTA; 10 mM) at pH = 7.0.

Relaxing solution: 3-(*N* morpholino)propanesulfonic acid (MOPS; 10 mM), potassium proprionate (64.4 mM), sodium sulfate (9.45 mM), magnesium proprionate (5.23 mM), potassium EGTA (2 mM), adenosine triphosphate (ATP; 7 mM) and creatine phosphate (10 mM) at pH = 7.0.

Activating solution: MOPS (10 mM), potassium proprionate (45.1 mM), magnesium proprionate (5.21 mM), sodium sulfate (9.27 mM), sodium EGTA (1 mM), ATP (7 mM), creatine phosphate (10 mM), calcium chloride (0.75 mM) at pCa = 3.12 and pH = 7.0.

## 3. Results

**Figure 2.**
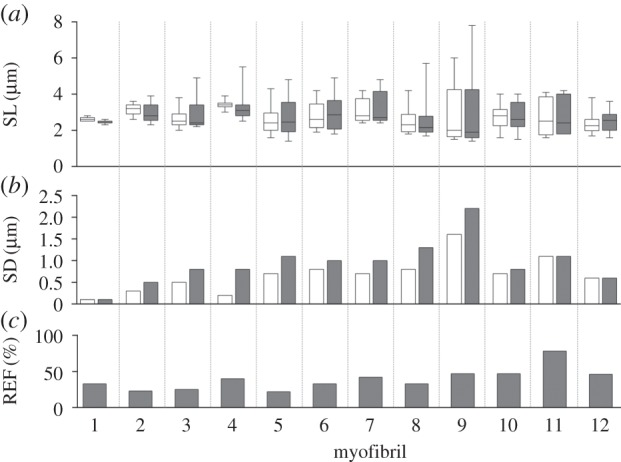
Results for each of the 12 myofibrils tested. (*a*) Tukey box plots of SLs for the I- (white) and FE- (shaded) states. (*b*) SD of the mean SL for the I- and FE-states. Note that a greater SD is indicative of a more non-uniform SL distribution. (*c*) Amount of RFE exhibited in each myofibril in the FE-state.

Individual myofibril results are presented in table 1, and visual representations are depicted in figure 2*a*–*c*. The I-state exhibited an average stress of 89 ± 35 nN µm^−2^ (mean ± 1 SD), which was significantly less than the average stress produced in the FE-state (121 ± 45 nN µm^−2^; *p* < 0.05), thereby exhibiting an average RFE of 39 ± 15%. The I-state stresses were comparable to values that have been observed previously [14,26,33,34,36,39].

**Table 1.** Summary of experimental variables for the 12 myofibrils tested. SL, SD and stress are given for both the I- and FE-states. Values were compared between states to identify the increase in non-uniformity (ΔSD) and amount of RFE.

		SL (μm)					stress			popped sarcomeres	
		I		FE							
myofibril (rabbit)	no. sarcomeres (*n*)	mean	SD	mean	SD	ΔSD (FE-I)	I (nN µm^−2^)	FE (nN µm^−2^)	RFE (%)	I (*n*)	FE (*n*)
1 (1)	10	2.6	0.1	2.5	0.1	0.0	59	78	33	0	0
2 (1)	13	3.1	0.3	3.0	0.5	0.2	132	162	23	0	0
3 (2)	11	2.7	0.5	2.9	0.8	0.3	126	158	25	0	1
4 (3)	23	3.4	0.2	3.3	0.8	0.6	74	104	40	0	4
5 (4)	16	2.5	0.7	2.7	1.1	0.4	94	114	22	1	3
6 (5)	14	2.8	0.8	3.0	1.0	0.2	87	115	33	2	3
7 (6)	5	3.1	0.7	3.2	1.0	0.3	35	50	42	1	1
8 (7)	8	2.5	0.8	2.6	1.3	0.5	100	134	33	1	1
9 (7)	9	2.9	1.6	3.0	2.2	0.6	87	129	47	2	2
10 (5)	13	2.7	0.7	2.8	0.8	0.1	58	86	47	1	1
11 (8)	5	2.7	1.1	2.8	1.1	0.0	59	105	78	1	1
12 (2)	10	2.4	0.6	2.5	0.6	0.0	151	220	46	0	0
mean ± SD		2.8 ± 0.3	0.7 ± 0.4	2.9 ± 0.3	0.9 ± 0.5	0.3 ± 0.2	89 ± 35	121 ± 45	39 ± 15		

### 3.1. Prediction 1

**Figure 3.**
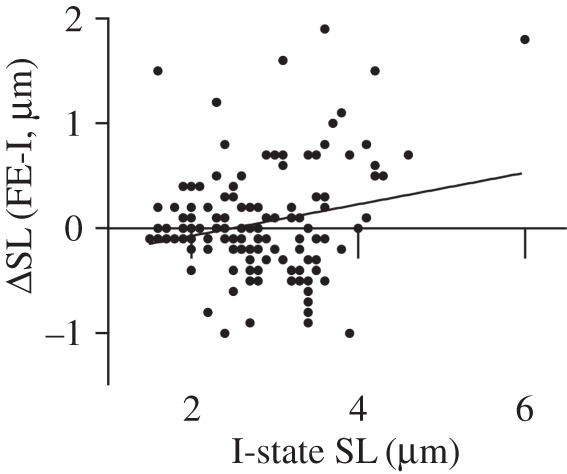
Change in individual SL from the I-state to the FE-state as a function of individual SL in the I-state for each of the 137 sarcomeres tested. The correlation between these variables was weak, but significant (*r* = 0.216; *p* = 0.011).

For the 137 individual sarcomeres tested within the 12 myofibrils, a weak but significant correlation was found between SL in the I-state and the length change (*r* = 0.216; *p* = 0.011; figure 3).

### 3.2. Prediction 2

Prior to myofibril activation SL non-uniformity was relatively small (mean SD = 0.1 µm). While there was a significant increase in non-uniformity (SD) from the I-state (0.7 ± 0.4 µm) to the FE-state (0.9 ± 0.5 µm; *p* < 0.05), three of the myofibrils (1, 11 and 12) did not demonstrate an increase in SL non-uniformity, one of which (myofibril 11) produced the greatest amount of RFE.

### 3.3. Prediction 3

The SLNT predicts that active stretching of muscle results in sarcomeres being stretched beyond actin–myosin filament overlap [24,25]. While a total of 17 popped sarcomeres were observed in nine of the 12 myofibrils in the FE-state, nine of these sarcomeres in seven myofibrils were also popped in the I-state. Therefore, popping of sarcomeres only accompanied the active stretch in four of 12 myofibrils (eight sarcomeres), whereas nine sarcomeres popped as a result of activation. In addition, three of the myofibrils produced RFE in the absence of popped sarcomeres. The Fisher's exact test revealed that there was no significant difference in the number of popped sarcomeres between the I- and FE-states (*p* = 0.148).

### 3.4. Prediction 4

**Figure 4.**
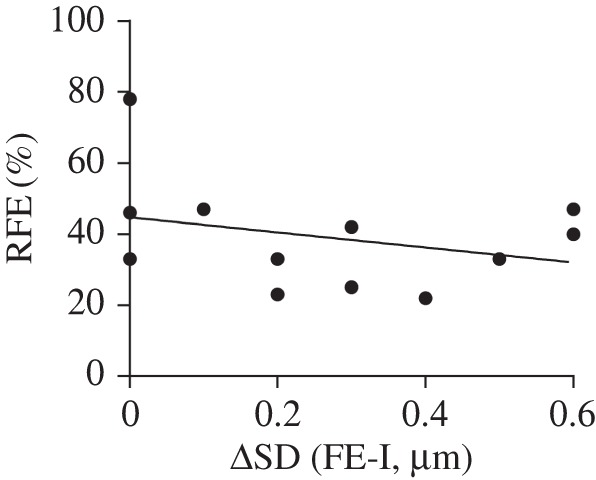
RFE as a function of the increase in SD from the I- state to the FE-state for all 12 myofibrils. There was no statistically significant relationship between these two variables for the conditions of these experiments (*r* = −0.309, *p* = 0.328).

There was no significant correlation between the amount of RFE exhibited by a myofibril and the increase in SL non-uniformity (*r* = −0.309, *p* = 0.328; figure 4).

## 4. Discussion

Here, we provide the first systematic comparisons of individual SL measurements between purely isometric reference contractions (I-state) and isometric contractions following active stretch (FE-state) in the same myofibril where individual SL and forces could be measured continuously. The results allowed for systematic testing of basic assumptions and predictions of the SLNT. All myofibrils exhibited substantial RFE, averaging 39% [9]. The first prediction of the SLNT that was tested was that shorter, stronger sarcomeres in the I-state will lengthen less than longer, weaker sarcomeres upon active stretch. While a significant relationship (*p* = 0.011) between the change in SL between states and SL in the I-state was found, we feel that the strength of this relationship (*r* = 0.216) and the variability of the individual data (figure 3) question the usefulness of this prediction. Specifically, figure 3 depicts a sarcomere that began relatively short (1.6 µm), but that increased in length by 1.5 µm following active stretch; and another sarcomere that began at a length just prior to popping (3.9 µm), but that shortened in length by 1.0 µm following active stretch. In addition to this, when the extreme outlier that had an initial SL of 6.0 µm was removed from analysis, the correlation was substantially lower and was no longer statistically significant (*r* = 0.122; *p* = 0.156). These data oppose the first prediction of the SLNT.

Despite a relatively large amount of non-uniformity present in the I-state, which has also been observed by other researchers [36], there was an increase in SL non-uniformity associated with active stretching of the myofibrils, which agrees with the second prediction of the SLNT [24,25]. However, three of the myofibrils showed RFE in the absence of an increase in SL non-uniformity, indicating that the development of SL non-uniformity is not necessary to achieve RFE in muscle, a finding that contradicts the SLNT. Myofibrils that did not show an increase in SL non-uniformity demonstrated a wide range of total non-uniformity, ranging from SL standard deviations of 0.1 to 1.1 µm.

The third prediction of the SLNT requires that sarcomeres must pop in order for RFE to occur [24,25]. While the majority of myofibrils (*n* = 9) exhibited popped sarcomeres in the FE-state, popping only accompanied the active stretch in four myofibrils. In the remaining five myofibrils, sarcomeres had already popped upon activation in the I-state at an average SL of about 2.8 µm. In addition, three myofibrils demonstrated RFE without popped sarcomeres. Force enhancement in the absence of popped sarcomeres has been observed previously [28]. However, in those experiments, an isometric steady state was not reached following active stretch. The finding that there was no significant difference in the number of popped sarcomeres between the I- and FE-states also suggests that sarcomere popping does not contribute to RFE in the manner proposed by the SLNT. Our results support earlier findings, which contradict the third prediction of the SLNT.

The lack of a significant correlation between the amount of RFE and the increase in non-uniformity, as well as the observation of RFE in the complete absence of an increase in SL non-uniformity suggests that while SL non-uniformities are often, but not always, present in the FE state, the amount of non-uniformity does not appear to influence the magnitude of the RFE. This finding contradicts the fourth prediction based on the SLNT.

It has been suggested that small variations in SLs along an isolated myofibril may be the result of differences in the number of contractile proteins [24], or the specific distribution of titin isoforms in adjacent sarcomeres [41,42]. Our experiments were not aimed at testing this possibility. Therefore, we cannot comment directly on the number of actin and myosin filaments, or the distribution of titin isoforms. However, if there were differences in the number of contractile proteins or structural elements between sarcomeres of a given myofibril, one would expect the resulting differences in SL to remain constant during our experiments. For example, the shortest sarcomere should always remain the shortest, and the longest sarcomere should always remain the longest. However, this was not the case as the order of SL changed with active stretching, an observation that has been made in previous experiments [14,18], thereby indicating that the SL non-uniformities observed in myofibrils [14,18,36], fibres [43] and whole muscles [23] are probably not caused by differences in the number or isoform of contractile or structural proteins.

There are limitations of this study that need to be kept in mind when interpreting the results. The three most critical ones are the fact that stretching of myofibrils was preceded by active shortening, the average SLs were not perfectly identical in the I- and FE-state, and that the experiments were performed on isolated myofibrils instead of *in vivo* skeletal muscles. Active shortening preceding active stretching, if anything at all, should have resulted in increased SL non-uniformities in the FE- compared with the I-state [17], therefore biasing our results in favour of the SLNT. In addition, the average SLs in the FE-state were on average 0.1 µm longer than in the I-state. Therefore, if anything at all, the RFE observed here are slight underestimates of the actual RFE, but again, this would not affect the general conclusions drawn from the findings of this study. The use of isolated myofibrils leads to a question of the generalizability of the data to what occurs within entire muscles *in vivo*. However, a previous study that observed sarcomere behaviour *in vivo* in passive and purely isometric contractions found approximately 20% variation in SL [23], which supports the current finding of 17% average variation in SL in the I-state. The FE-state exhibited an average variation of 24%, which we feel is also within reasonable proximity to the *in vivo* data. Therefore, it appears that SL non-uniformities observed in our isolated myofibril preparations agree well with those in whole muscle preparations. This agreement of course does not imply that results from myofibrils can be generalized to entire muscles, but it lends credence to the idea that similar observations to those made here would probably be made in whole muscle preparations. However, this will need to be confirmed with experiments where active stretching can be accomplished while simultaneously following a number of sarcomeres *in situ*. Such a study will require technical developments, as nobody has been able to make such measurements to date. Finally, the variability of the results regarding SL distributions and amount of RFE, despite the rigorous and standardized experimental protocol, leads to the conclusion that the phenomenon of RFE cannot be solely credited to the mathematical explanation provided by the SLNT.

## 5. Conclusion

Based on the findings of this study, we conclude that RFE is not simply a product of the development of SL non-uniformities during active stretch in the manner proposed by the SLNT. Our results oppose the mathematical basis of the SLNT. We observed RFE in the absence of an increase in SL non-uniformities from I- to FE-states and in the absence of popped sarcomeres [24,25]. SL non-uniformity was primarily observed upon activation of myofibrils, and not upon active stretching, and thus appears to be a natural part of muscle contraction, as has been observed in fully intact muscles and muscle fibres [23,43].

## Supplementary Material

“The Role of Sarcomere Length Non-Uniformity in Residual Force Enhancement of Skeletal Muscle Myofibrils - Data.xlsx” This file contains the experimental dataset from 12 isolated skeletal muscle myofibrils testing the sarcomere length non-uniformity theory, which attempts to explain the phenomenon of residual force enhancement. Each sheet within the excel file corresponds to one of the myofibrils that was tested. There are force and individual sarcomere length data for each time point throughout the experiment. The final sheet in the file provides the diameters for each of the myofibrils.

## Data accessibility

Data supporting this manuscript have been uploaded as part of the electronic supplementary material.
